# Cardiovascular hemodynamics in mice with tumor necrosis factor receptor—associated factor 2 mediated cytoprotection in the heart

**DOI:** 10.3389/fcvm.2023.1064640

**Published:** 2023-05-09

**Authors:** Andrea G. Marshall, Kit Neikirk, Zer Vue, Heather K. Beasley, Edgar Garza-Lopez, Larry Vang, Taylor Barongan, Zoe Evans, Amber Crabtree, Elsie Spencer, Josephs Anudokem, Remi Parker, Jamaine Davis, Dominique Stephens, Steven Damo, Thuy T. Pham, Jose A. Gomez, Vernat Exil, Dao-fu Dai, Sandra A. Murray, Mark L. Entman, George E. Taffet, Antentor O. Hinton, Anilkumar K. Reddy

**Affiliations:** ^1^ Department of Molecular Physiology and Biophysics, Vanderbilt University, Nashville, TN, United States; ^2^ Department of Internal Medicine, University of Iowa, Iowa City, IA, United States; ^3^ Department of Biochemistry and Cancer Biology, Meharry Medical College, Nashville, TN, United States; ^4^ Department of Life and Physical Sciences, Fisk University, Nashville, TN, United States; ^5^ Department of Medicine, Baylor College of Medicine, One Baylor Plaza, Houston, TX, United States; ^6^ Department of Medicine, Vanderbilt University Medical Center, Nashville, TN, United States; ^7^ Department of Pediatrics, Div. of Cardiology, St. Louis University School of Medicine, St. Louis, MO, United States; ^8^ Department of Pediatrics, Carver College of Medicine, University of Iowa, Iowa City, IA, United States; ^9^ Department of Pathology, Carver College of Medicine, University of Iowa, Iowa City, IA, United States; ^10^ Department of Cell Biology, College of Medicine, University of Pittsburgh, Pittsburgh, United States

**Keywords:** systolic and diastolic function, myocardial performance index, ventricular-vascular coupling, arterial and left ventricular elastance, aortic impedance

## Abstract

**Introduction:**

Many studies in mice have demonstrated that cardiac-specific innate immune signaling pathways can be reprogrammed to modulate inflammation in response to myocardial injury and improve outcomes. While the echocardiography standard parameters of left ventricular (LV) ejection fraction, fractional shortening, end-diastolic diameter, and others are used to assess cardiac function, their dependency on loading conditions somewhat limits their utility in completely reflecting the contractile function and global cardiovascular efficiency of the heart. A true measure of global cardiovascular efficiency should include the interaction between the ventricle and the aorta (ventricular-vascular coupling, VVC) as well as measures of aortic impedance and pulse wave velocity.

**Methods:**

We measured cardiac Doppler velocities, blood pressures, along with VVC, aortic impedance, and pulse wave velocity to evaluate global cardiac function in a mouse model of cardiac-restricted low levels of TRAF2 overexpression that conferred cytoprotection in the heart.

**Results:**

While previous studies reported that response to myocardial infarction and reperfusion was improved in the TRAF2 overexpressed mice, we found that TRAF2 mice had significantly lower cardiac systolic velocities and accelerations, diastolic atrial velocity, aortic pressures, rate-pressure product, LV contractility and relaxation, and stroke work when compared to littermate control mice. Also, we found significantly longer aortic ejection time, isovolumic contraction and relaxation times, and significantly higher mitral early/atrial ratio, myocardial performance index, and ventricular vascular coupling in the TRAF2 overexpression mice compared to their littermate controls. We found no significant differences in the aortic impedance and pulse wave velocity.

**Discussion:**

While the reported tolerance to ischemic insults in TRAF2 overexpression mice may suggest enhanced cardiac reserve, our results indicate diminished cardiac function in these mice.

## Introduction

Tumor Necrosis Factor (TNF) Receptor Associated Factor 2 (TRAF2), an E3 Ubiquitin-protein ligase, interacts with signaling molecules responsible for cell death, cellular stress response, activation of nuclear factor-*k*B and JNK (1, 2) and critical pathways for cellular senescence and regeneration (3). The TRAF2 pathway is activated by tumor necrosis factor and recruits for apoptosis inhibitor proteins, cIAP1/2 (4). Tumor necrosis factor can protect against ischemic-induced cardiomyocyte death (5). In contrast, its knockout is less cardioprotective (6, 7). In cancer studies, it was shown that TRAF2 can act as both a tumor promotor and suppressor (8). Given such juxtaposing roles, it is not surprising that mixed outcomes have been reported on the role of TRAF2 as a potential downstream mediator of cardiac protection in heart failure. TNF is cytoprotective in the heart *via* TRAF2-mediated activation of NF-kB (9), with TRAF2 mitochondrial localization being cytoprotective and potentially contributing to TRAF2-mediated mitophagy (10). Beyond this, TRAF2 mediates myocardial survival by suppressing apoptosis and necroptosis (11). These roles make TRAFs important targets in many inflammatory and autoimmune diseases (12). However, other studies have suggested that cardiac function was improved after myocardial infarction in absence of NF-kB p50 subunit (13), showing that suppressing NF-kB with oligonucleotide decoys reduces ischemia-reperfusion (IR) myocardial injury (14) and TRAF2 mediates enhanced cardiac hypertrophy *via* activation of AKT/GSK3*β* signaling (15). Regardless, overexpression of TRAF2 was found to have a cardioprotective role in myocardial injury and perhaps can be a novel therapeutic strategy in cardiomyopathy (16).

Most studies in mice use echocardiography measurements of LV ejection fraction (EF), fractional shortening (FS), and end-diastolic diameter (EDD), among others, to assess cardiac function much akin to those used in clinical studies (17). However, EF, FS, or EDD are dependent on loading conditions and not completely reflective of the contractile function of the heart (18). Therefore, it is important to include the interaction between the ventricle and the aorta (also known as ventricular-vascular coupling, VVC) to evaluate the performance of LV as a true measure of global cardiovascular efficiency (17, 19). The VVC, as determined as the ratio of aortic elastance (Ea) and end-systolic elastance (Ees), has recently been recommended for risk stratification for heart failure (20). However, the Ea and Ees parameters may be less sensitive in disease, when more sensitive parameters of myocardial longitudinal strain and aortic pulse wave velocity or aortic impedance may be used (20, 21).

In their study, Burchfield et al. (9) mainly characterized the cardiac function to evaluate the effects of cardiac-restricted low levels of TRAF2 overexpression (MHC-TRAF2_LC_) in mice and concluded that TNF is cytoprotective in the heart *via* TRAF2 mediated activation of NF-kB. We, however, contend that such models should be studied holistically by evaluating their global cardiovascular function using measurements such as VVC and aortic impedance to determine true global myocardial performance. The goal of this study was to comprehensively evaluate the baseline global cardiovascular function of the same TRAF2 mouse model in comparison to their age-matched littermates using cardiac systolic, diastolic, aortic and LV pressure, VVC, and aortic impedance.

## Methods

### Animals

**Figure 1:**
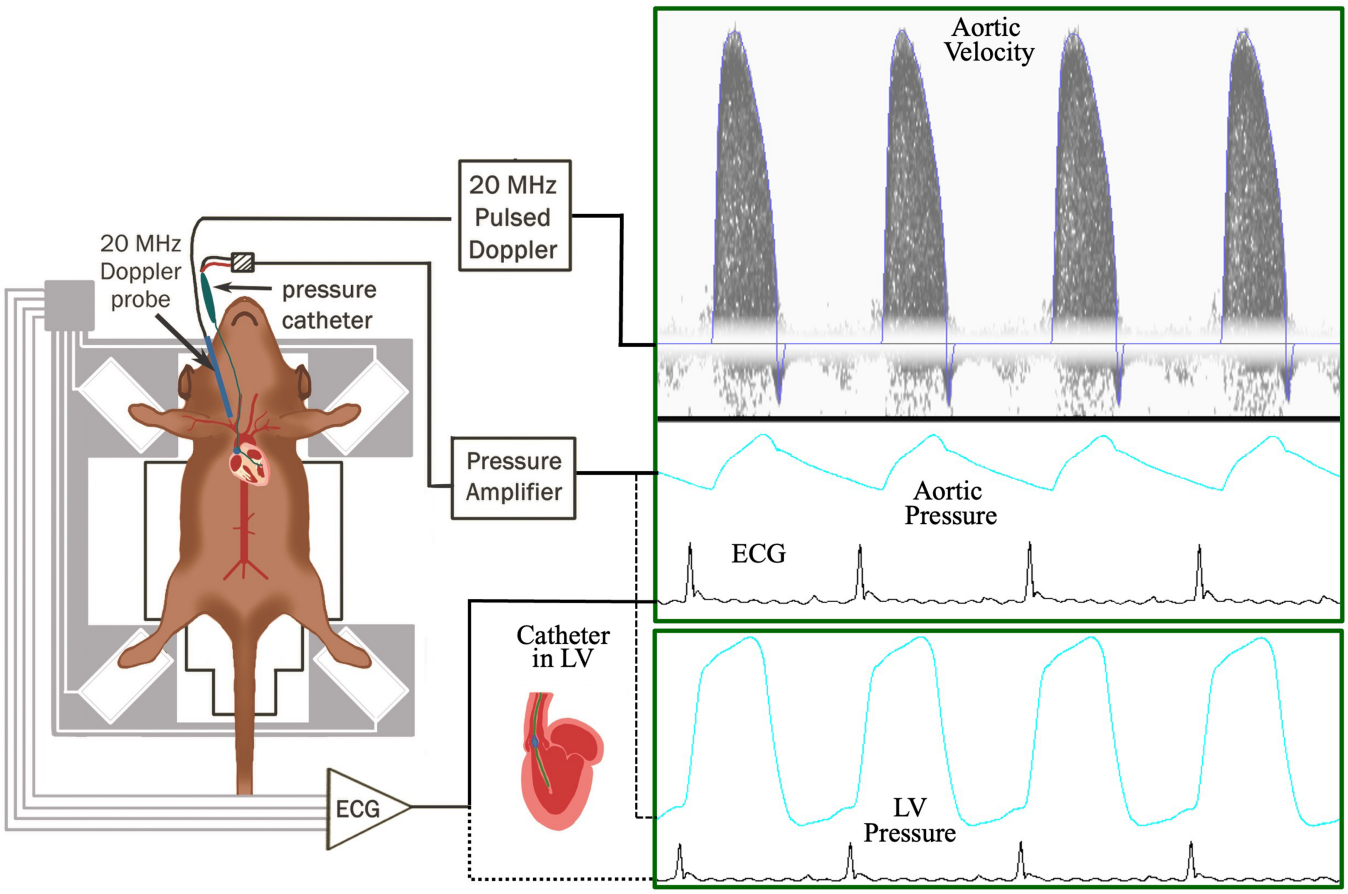
Workflow of the experimental setup to measure aortic blood flow velocity, aortic blood pressure, and ECG (electrocardiogram) in TRAF2 and control mice.

The generation of MHC-TRAF2_LC_ mice was previously described (9). Briefly, animals were generated with *α*-myosin heavy chain promoter targeting murine TRAF2 and characterization of the mice was done using histological analysis at 12 weeks (9). We used 8 male MHC-TRAF2_LC_ at 2–3 months of age. The diets of both groups of mice consisted of standard commercial chow (2,920× Harlan Teklad, Indianapolis, IN, USA) with free access to food and water. All animal protocols were approved by the Institutional Animal Care and Use Committee of Baylor College of Medicine in accordance with the National Institutes of Health Guide for the Care and Use of Laboratory Animals. Anesthesia was initially induced in mice with 2.5% isoflurane before transfer to a heated (37 ± 1°C) electrocardiography board (MouseMonitor S, Indus Instruments, Webster, TX) with isoflurane maintained at 1.5%. The mouse paws were attached to electrodes to measure electrocardiogram (ECG). The experimental setup and the workflow is shown in Figure 1.

### Doppler flow velocity measurements

Cardiac Doppler aortic outflow velocity and mitral inflow signals were measured with 20 MHz Doppler probe with all signals (including blood pressure and ECG) simultaneously acquired using Doppler Flow Velocity System (DFVS; Indus Instruments, Webster, TX). From the cardiac Doppler signals, we calculated peak and mean aortic velocities, stroke distance (Sd), aortic ejection time (ET), peak and mean aortic accelerations, Mitral early peak (E) and atrial peak (A) velocities, E/A ratio, E-deceleration time (DT), isovolumic contraction (IVCT) & relaxation (IVRT) times, and myocardial performance index (also known as Tei index = (IVCT + IVRT)/ET).

### Blood pressure and flow velocity measurements

Blood pressure measurements were made as previously described (22, 23). Briefly, the right carotid artery was isolated and cannulated with a pressure catheter (SPR-1,000: Millar Instruments, Inc., Houston, TX) and advanced into the ascending aorta to measure aortic pressure (along with simultaneous measurement of aortic blood flow velocity as described below). The aortic pressure and velocity signals were displayed simultaneously, and 2 s segments of both signals were recorded using DFVS system. The catheter was then advanced into the LV. Again, 2 s segments of LV pressure, and systolic (SBP), diastolic (DBP), mean (MBP), pulse pressures (PP), and rate-pressure product (RPP) were calculated from aortic pressure. Peak LV pressure (P_LVP_), indices of contractility (+dP/dt**_max_**) and relaxation (−dP/dt**_max_**), relaxation time constant (tau), and LV end-diastolic pressure (LVEDP) were calculated from LV pressure.

### Determination of aortic input impedance

Aortic input impedance was determined using previously reported methods (22–25). Briefly, impedance was determined using the simultaneously measured ascending aortic pressure and velocity signals. The pressure and velocity waveforms were processed using a discrete Fourier transformation to determine aortic input impedance (Zi). Peripheral vascular resistance (Zp—impedance at zero frequency), the impedance at first harmonic (Z_1_), and characteristic impedance (Zc—an average of 2nd to 10th harmonic) were extracted from the modulus of aortic impedance (|Zi|). Pulse wave velocity was calculated from |Zi| as Zc/*ρ* (ρ—density of blood).

### Calculation of parameters to determine ventricular-vascular coupling

Arterial elastance (Ea) was calculated as ESP/SV (stroke volume, SV = Sd * aortic cross-sectional area), end-systolic elastance (Ees) was calculated as ESP/ESV, ventricular-vascular coupling (VVC) was calculated as Ea/Ees, and stroke work (SW) was calculated as ESP*SV (17).

### Statistical analyses

All the data are presented as mean ± standard error of the mean (SEM). Statistical analyses were performed *via* analysis using Student's T-test on Prism (GraphPad Software; La Jolla, USA). Non-parametric tests (Mann-Whitney, in lieu of t-test) were utilized for data that were not normally distributed, as determined by the Kolmogorov-Smirnov, Shapiro-Wilk, Anderson-Darling, and D'Agostino & Pearson tests. Normalcy is indicated in Supplementary Figure S1 as QQ plots with non-normally distributed data marked by *.

## Results

**Figure 2:**
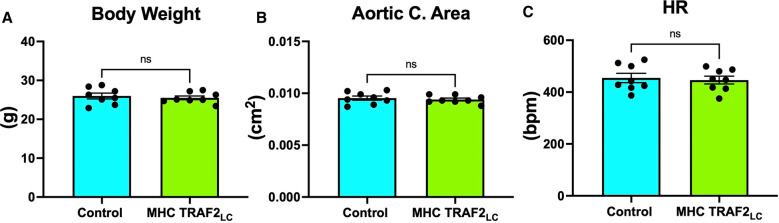
General parameters of male control mice and MHC-TRAF2_LC_ mice. (**A**) Body weight in grams, (**B**) aortic cross-sectional area, and (**C**) heart rate (HR) in beats per minute are shown. Data are presented as mean ± SEM (*n* = 8/group); ns indicates a statistically non-significant relationship.

**Figure 3:**
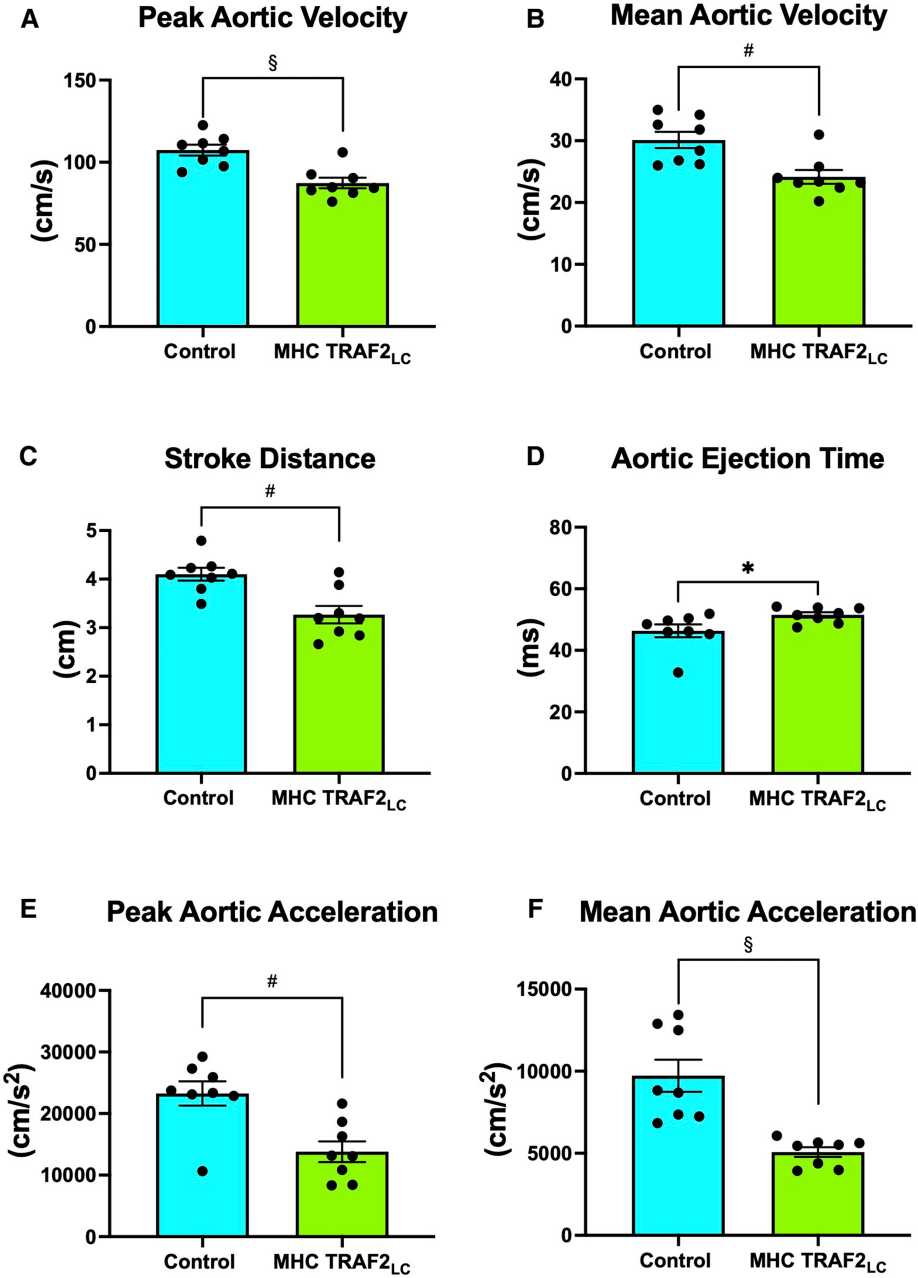
Aortic flow velocity indices of male control mice and MHC-TRAF2_LC_ mice. (**A**) Peak aortic velocity, (**B**) mean aortic velocity, (**C**) stroke distance, (**D**) aortic ejection time, (**E**) peak aortic acceleration, and (**F**) mean aortic acceleration. Data are presented as mean ± SEM (*n* = 8/group). *,#, and § represents *p* < 0.05, *p* < 0.01, and *p* < 0.001, respectively.

The general physiological parameters were measured in TRAF2 mice and compared to CTR mice. Body weight, aortic cross-sectional area, and heart rate (HR) were unaffected by TRAF2 overexpression (Figures 2A–C). We determined the impact of TRAF2 overexpression on cardiac systolic function and found a significant decrease in peak aortic velocity, mean aortic velocity, and stroke distance in TRAF2 mice compared to CTR mice (Figures 3A–C). We also found a significant increase in aortic ejection time (ET) (Figure 3D) and significant decreases in both peak and mean acceleration in TRAF2 mice (Figures 3E, F).

**Figure 4:**
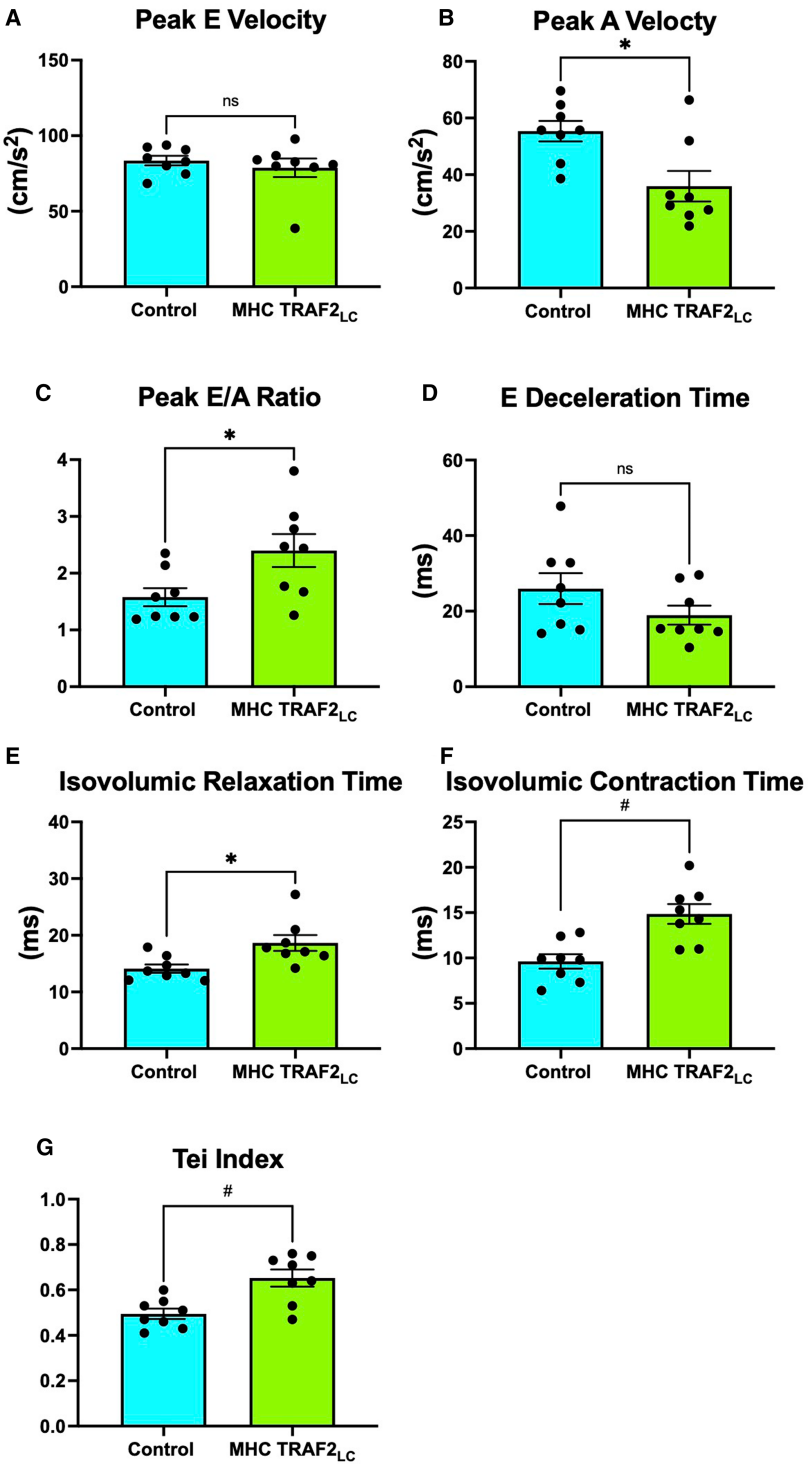
Mitral flow velocity indices of male control mice and MHC-TRAF2_LC_ mice. (**A**) peak mitral-early (**E**) flow velocity, (**B**) peak mitral-atrial (**A**) flow velocity, (**C**) mitral E/A ratio, (**D**) E-deceleration time, (**E**) isovolumic relaxation time, (**F**) isovolumic contraction time, and (**G**) myocardial performance index (Tei index), a measure of combined diastolic and systolic function, determined by the sum of isovolumetric relation and contraction time normalized against ejection time. All measurements obtained from cardiac Doppler flow velocity signals in mice. Data are presented as mean ± SEM (*n* = 8/group). * and # represents *p* < 0.05 and *p* < 0.01, respectively. Ns indicates statistically non-significant relationship.

While there were no significant differences in mitral peak early velocity (E), peak atrial velocity (A) decreased significantly, resulting in the near doubling of the Peak E/A ratio (Figures 4A–C). We also observed a significant decrease in E-deceleration time and significant increases in both isovolumetric relaxation (IVRT) and contraction time (IVCT) with TRAF2 overexpression (Figures 4D–F). Myocardial performance index (MPI, also known as Tei Index) increased significantly in TRAF2 mice compared to CTR mice (Figure 4G).

**Figure 5:**
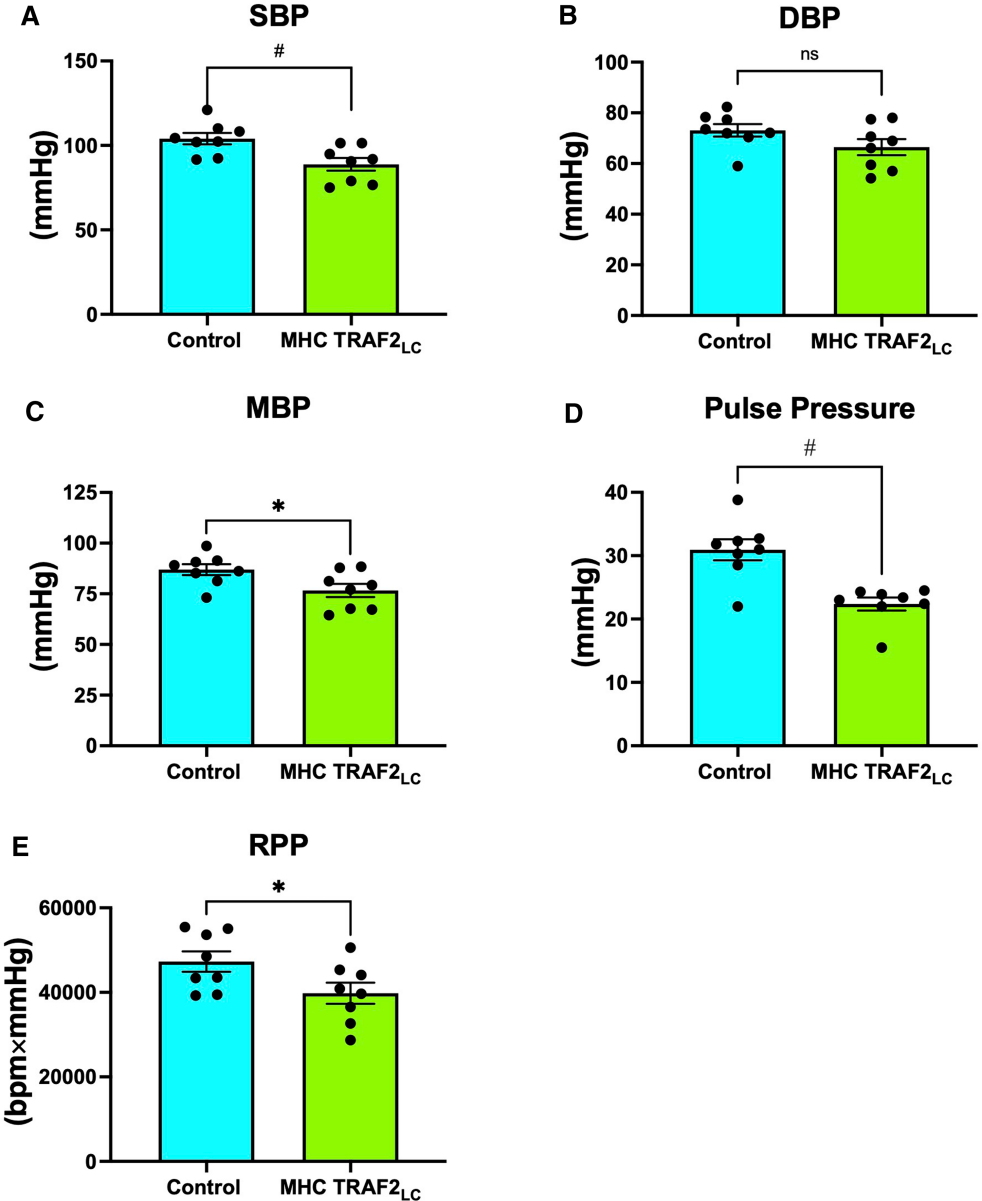
Aortic blood pressure and rate pressure product of male control mice and MHC-TRAF2_LC_ mice. (**A**) Systolic blood pressure (SBP), (**B**) Diastolic blood pressure (DBP), (**C**) Mean blood pressure (MBP), (**D**) Pulse pressure, and (**E**) Rate pressure product (RPP). Data are presented as mean ± SEM (*n* = 8/group). *,#, and § represents *p* < 0.05, *p* < 0.01, and *p* < 0.001, respectively; ns indicates statistically non-significant relationship.

Systolic blood pressure (SBP) showed a significant decrease with TRAF2 overexpression, while diastolic blood pressure (DBP) showed a slight decrease, but it was not statistically significant (Figures 5A, B). Furthermore, mean blood pressure (MBP), pulse pressure (PP = SBP-DBP), and rate pressure product (RPP = SBP * HR) all decreased significantly in TRAF2 mice compared to the CTR mice (Figures 5C–E).

**Figure 6:**
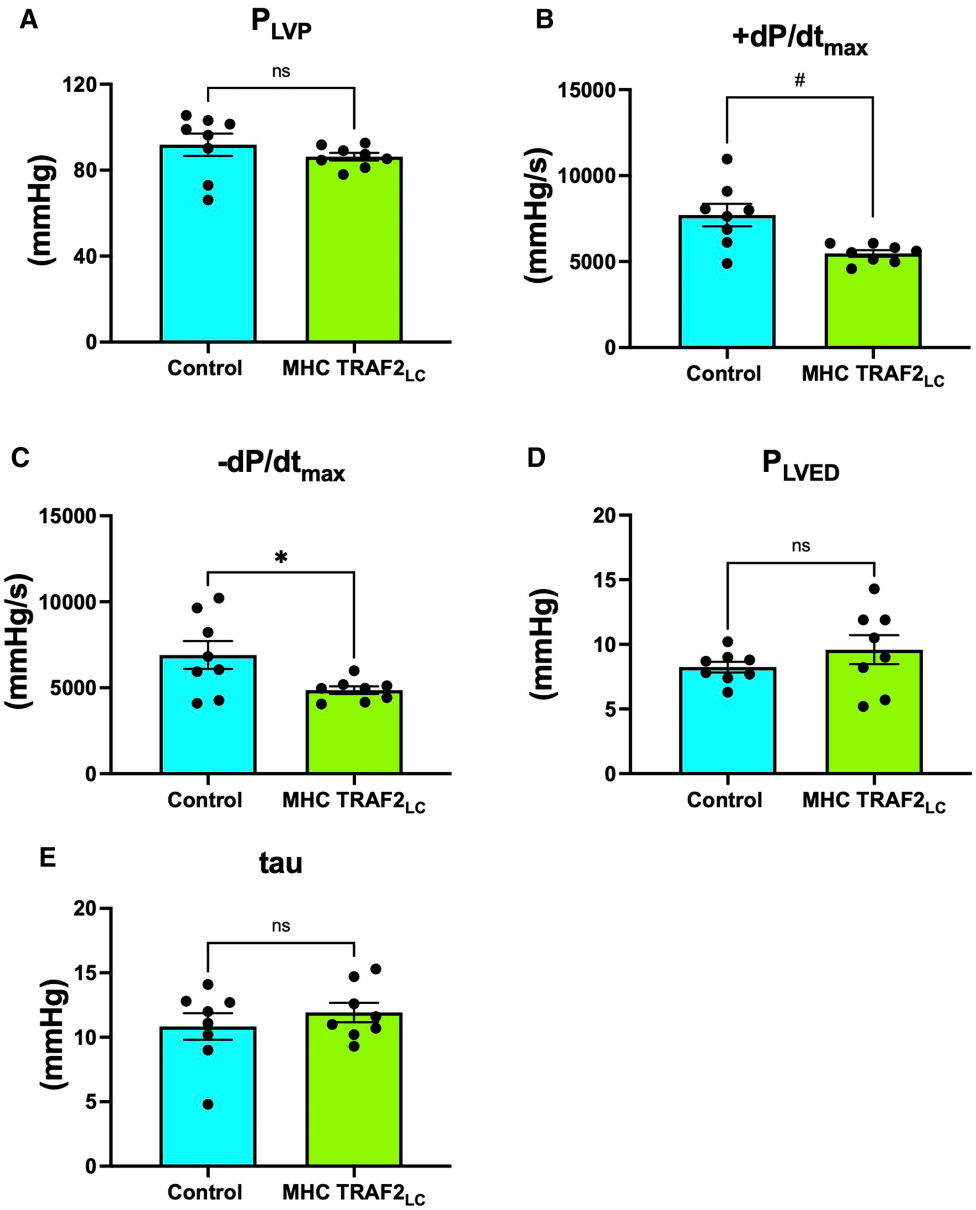
Left ventricular blood pressure parameters of male control mice and MHC-TRAF2_LC_ mice. (**A**) Peak left ventricular pressure (P_LVP_), (**B**) Maximal contractility (+dP/dt_max_), (**C**) Maximal relaxation (-dP/dt_max_), (**D**) left ventricular end diastolic pressure (P_LVED_), and (**E**) relaxation time constant (tau). Data are presented as mean ± SEM (*n* = 8/group). * and # represents *p* < 0.05 and *p* < 0.01, respectively; ns indicates statistically non-significant relationship.

While peak left ventricular pressure (P_LVP_) was not significantly different from CTR mice (Figure 6A), both + dP/dt_max_ and −dP/dt_max_ were significantly lower in TRAF2 mice compared to CTR mice (Figures 6B, C). The relaxation time constant (tau) and LV end-diastolic pressure (P_LVED_) trended higher but were not significantly different in TRAF2 mice compared to CTR mice (Figures 6D, E).

**Figure 7:**
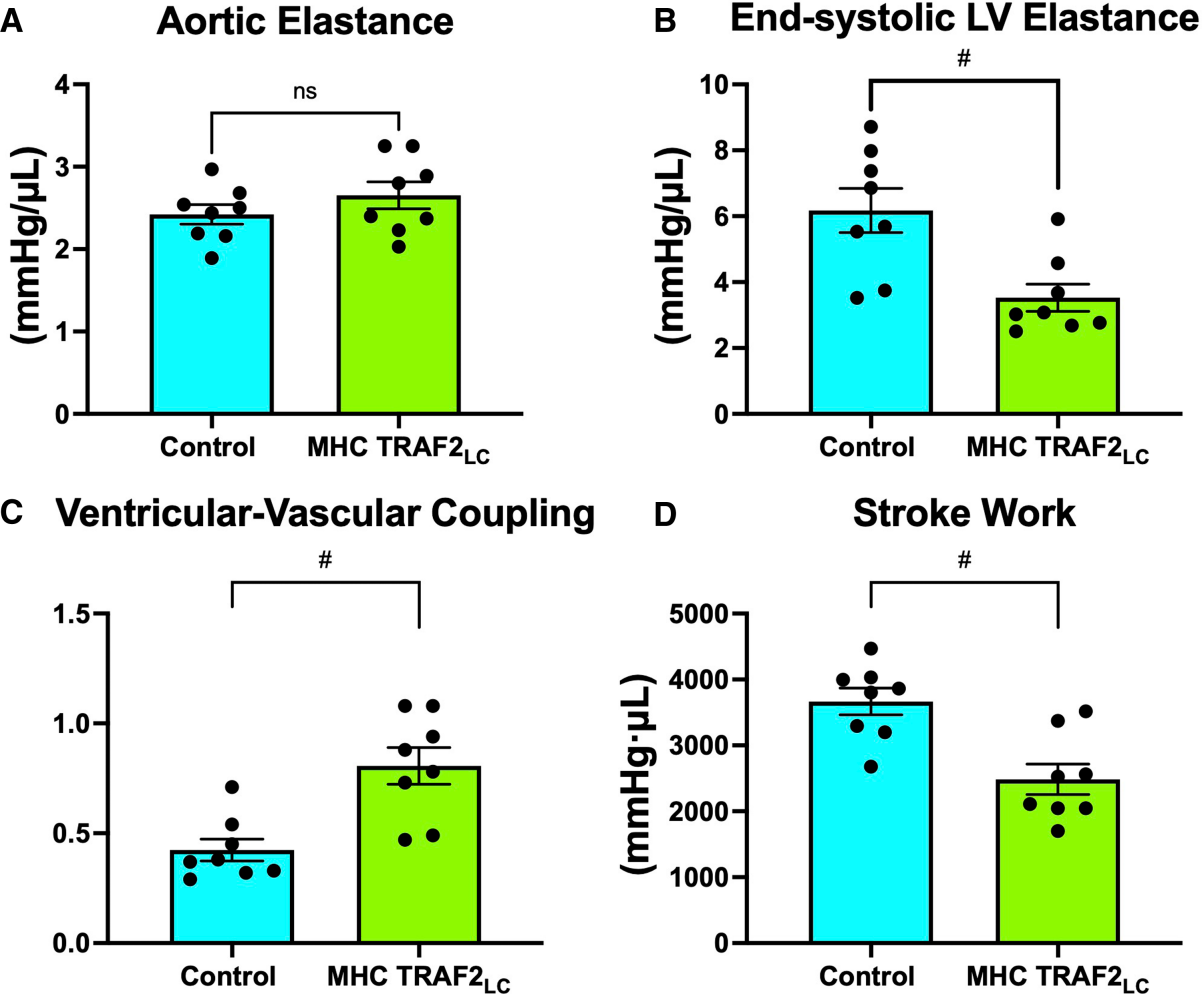
Parameters of ventricular-vascular coupling. (**A**) aortic elastance, (**B**) end-systolic LV elastance, (**C**) ventricular-vascular coupling, and (**D**) stroke work. # represents *p* < 0.01 and ns indicates not statistically significant.

There were no differences in arterial elastance (Ea) but end-systolic LV elastance (Ees) was significantly lower in TRAF2 mice, resulting in ventricular-vascular coupling being significantly higher when compared to controls (Figures 7A–C). Also, stroke work was significantly lower in the TRAF2 mice when compared to CTR mice (Figure 7D).

**Figure 8:**
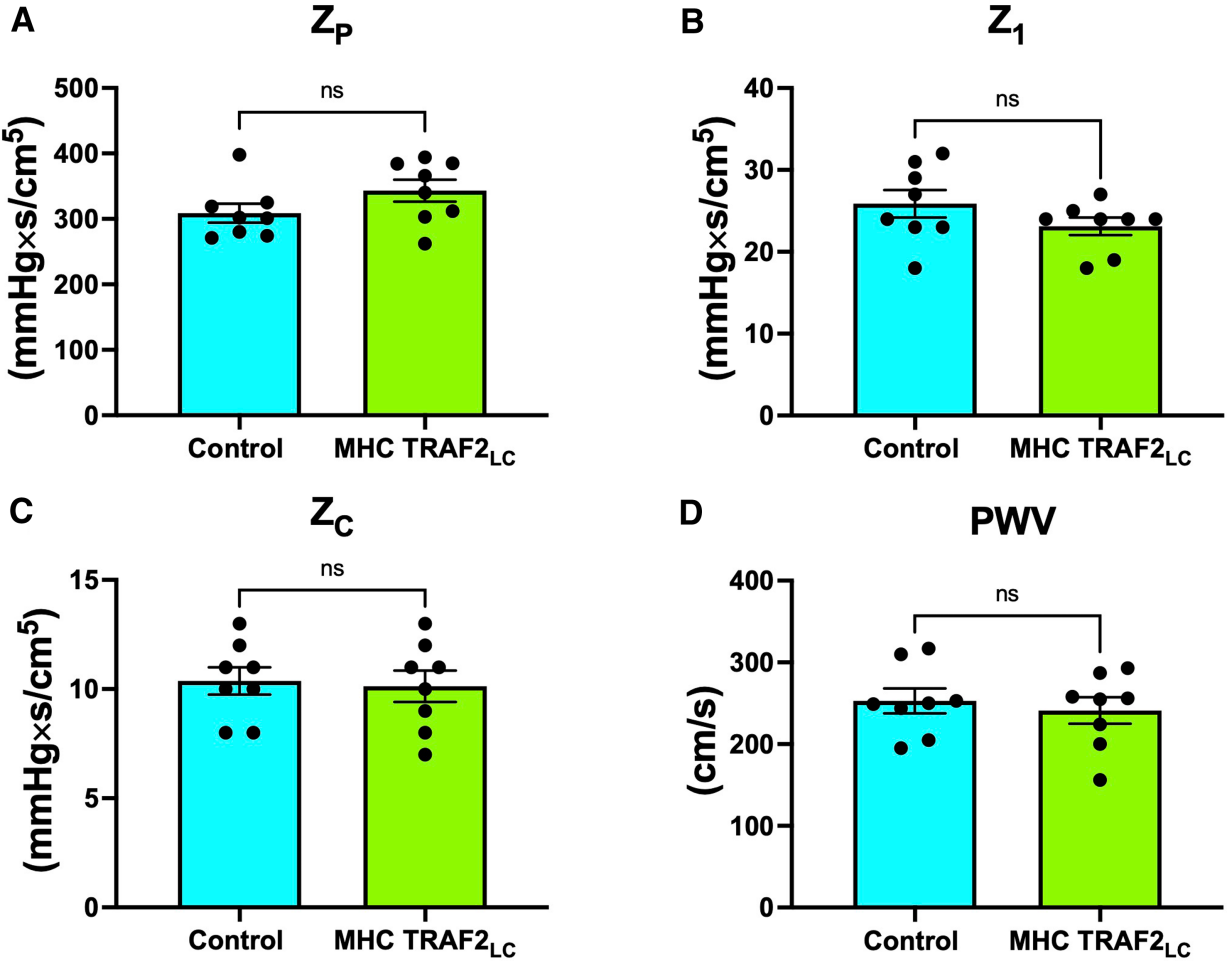
Parameters of aortic impedance in male control mice and MHC-TRAF2_LC_ mice. (**A**) Total peripheral resistance (Z_P_), (**B**) impedance at first harmonic (Z_1_), (**C**) characteristic impedance, (Z_C_) (**D**), and impedance-based pulse wave velocity (PWV_Z_). Data are presented as mean ± SEM (*n* = 8/group); ns indicates statistically non-significant relationship.

LV afterload was evaluated using aortic impedance. We found no significant differences in the two groups' impedance parameters, Zp, Z1, Zc, or PWVz (Figures 8A–D).

## Discussion

A previous study by Burchfied et al. (9) suggested a TRAF2-mediated cardioprotective function, as mainly evaluated in the heart. But such models should be studied by evaluating the *in situ* global cardiovascular function. In this study, we used invasive measurements of aortic and LV pressure, noninvasive measurements of Doppler mitral inflow and aortic flow velocity, and the calculated parameters of VVC and aortic impedance to evaluate the cardiac and aortic hemodynamics in TRAF2 mice compared to their age-matched littermates to determine true global myocardial performance. The general parameters of body weight, aortic cross-sectional area, and heart rate of TRAF2 overexpressing mice were not significantly different from CTR mice (Figure 2).

### Cardiac systolic and diastolic function

We found that the cardiac systolic function, as determined by peak and mean aortic flow velocities, peak, and mean aortic accelerations (Figure 3) and + dP/dt_max_ (Figure 4), is diminished in TRAF2 mice compared to the CTR mice, which may indicate a mild systolic dysfunction or enhanced systolic reserve. The LV pressure indices of contractility (+dP/dt_max_) and relaxation (−dP/dt_max_) were significantly lower in the TRAF2 mice and followed a similar trend that was observed in the aortic blood pressure and velocity, and in agreement with the decrease in %FS reported by Burchfield et al. (9) Again, these diminished contractile and relaxation functions in TRAF2 mice are similar to what we observed in the young dwarf *Little* mice (26). We have not challenged these mice to determine systolic reserve capacity, but our group showed that left ventricular developed pressure recovered better in the TRAF2 mice after IR injury and observed that the cytoprotective effects of TRAF2 were mediated by NF-*κ*B activation (9). However, others have reported that suppression of NF-kB with oligonucleotide transcription factor decoys diminishes IR myocardial injury (14) and that cardiac function improved after myocardial infarction with the absence of NF-kB p50 unit (13). Our group reiterated the cytoprotective effects of TRAF2 may have been afforded *via* crosstalk between canonical and noncanonical NF-kB signaling pathways (27). Other groups have also shown that silencing of TRAF2 results in the dysregulation of NF-*κ*B marked by overactivation (28). Conversely, TRAF2 overexpression has been linked to the activation of I*κ*B kinase, responsible for the nuclear translocation of NF-*κ*B (29). Given the relevance of NF-*κ*B in developing an anti-inflammation drug, future studies must further expound upon the changes in NF-*κ*B upon TRAF2 expression, as activation of the NF-*κ*B offers a potential pathway by which TRAF2 overexpression mediates cardiac function.

A more recent study noted that TRAF2 maintains myocardial homeostasis through the facilitation of cardiac myocyte mitophagy thus preventing inflammation and cell death (16). We also found that aortic ejection times were significantly prolonged in the TRAF2 mice (Figure 3D). Obata et al. (30) reported that longer aortic ejection time is associated with lower blood pressures (30), and perhaps the low blood pressure in TRAF2 mice in our study may have caused longer ejection times. Also, longer ET results from longer action potential duration in old animals and may be associated with cellular changes in the mechanics of excitation–contraction coupling (31). Similar cellular changes may be present in TRAF2 that could have caused prolonged ejection time.

The cardiac diastolic function, as defined by E/A ratio, was higher in TRAF2 mice than CTR mice (Figure 4C) indicating improved diastolic function. However, E >> A (or E/A > 2) and shorter E-deceleration time is associated with a moderate decrease in compliance (32). The TRAF2 mice had E/A > 2 and shorter E-deceleration time (Figure 4D) perhaps indicating a decline in compliance which agrees with the observation by Burchfield et al. (9), that TRAF2 mice had mildly hypertrophic hearts (9). Isovolumic contraction time is the index of LV contractility and isovolumic relaxation time is an index of LV relaxation, but both are dependent on the heart rate (33) and other factors. Prolonged IVRT indicates impaired relaxation and prolonged IVCT is associated with reduced EF, both increasing the risk for heart failure (34). Since IVCT and IVRT are both associated with LV ejection time, the myocardial performance index (MPI or Tei Index) provides an overall cardiac performance (33). We found that TRAF2 overexpression resulted in a small but significant increase in Tei Index (Figure 4G), which may indicate impaired cardiac function like that reported in patients (35).

While TRAF2 overexpression confers cardioprotective effects, the small but significant changes in the systolic and diastolic parameters indicate a modest decrease in baseline function. It remains to be seen if this diminished function deteriorates with age or remains stable over their life span like that in the dwarf *Little* mice, which maintain a diminished but stable cardiac function over their life span (26).

### Aortic and left ventricular pressure

TRAF2 mice had significantly lower systolic, mean, and pulse pressures compared to CTR mice. The lower blood pressures combined with lower blood velocities in TRAF2 mice make LV afterload appear normal. Previous studies reported that every 20 or 10 mmHg increase in SBP or DBP, respectively, beyond traditionally healthy values (36) doubles the chance of major long-term consequences, such as heart failure or stroke (37). Since SBP remains a stronger indicator of cardiovascular health than DBP at ages over 50, our findings suggest that the cardioprotective effects of TRAF2 potentially may be conferred across aging (38). Pulse pressure that is ≤25% of systolic pressure is considered too low and typically occurs in aortic stenosis, cardiac tamponade, aortic stenosis, or with blood loss (39). Pulse pressure in TRAF2 mice was ≈25% of systolic pressure, perhaps indicating modest dysfunction. Given that we measured blood pressure in these mice at a young age, studies at older ages may reveal if the cardioprotective effects of TRAF2 overexpression are maintained across the lifespan. The rate pressure product (RPP = SBP × heart rate), which is an indirect measurement of the heart oxygen consumption (40), was significantly lower in TRAF2 mice than in CTR mice, indicating a lower heart workload.

### Ventricular-vascular coupling

A measure of myocardial performance that includes interaction with aortic function, the ventricular-vascular coupling (VVC) was significantly higher in the TRAF2 mice indicating a diminished global cardiovascular efficiency. While aortic elastance was unchanged, the higher VVC in TRAF2 mice was mainly due to significantly lower end-systolic LV elastance. Similar observations were made in patients with major adverse cardiovascular events (17). Also, it has been shown that decreased LV stroke work index is associated with diminished LV systolic and diastolic function in cardiac intensive care unit patients (41). We found that stroke work is significantly lower in the in the TRAF2 mice, perhaps indicating a diminished cardiac function.

### Aortic impedance

Defined as the ratio of modulus of pressure to modulus of velocity at several harmonics (0–10) in the frequency domain, aortic impedance provides a comprehensive description of the afterload experienced by the left ventricle. This includes the pulsatile and the steady components of the hydraulic load compared with when pressure and velocity are considered individually (23, 24, 42). We used blood flow velocity instead of volume flow because both blood flow velocity and pressure are independent of body weight or size (24). We found that the peripheral vascular resistance (Zp, impedance modulus at zero frequency), the strength of wave reflections from the periphery (Z1, impedance modulus at the first harmonic), and characteristic impedance (Zc, average of 2–10 harmonics of impedance modulus), and pulse wave velocity (PWVZ; derived from Zc; aortic stiffness index) were not different in TRAF2 mice when compared to CTR mice (Figure 8). The unchanged Zc & PWV_Z_ seems to conflict with our finding that pulse pressure was lower in TRAF2 mice. While PWV is a direct measure of aortic stiffness, pulse pressure is used as a surrogate stiffness index that also reflects stroke volume (which was also lower in TRAF2 mice) and both measures (stiffness & pulse pressure) are mildly correlated (43). In another study anti-hypertensive treatments in patients resulted in no changes in aortic PWV but had a significant decrease in brachial pulse pressure (44).

## Limitations

These observations are made in these mice at a young age and the effectiveness of TRAF2-mediated cytoprotection of the heart and cardiovascular system with age needs to be investigated. 2–3 month mice were chosen due to establishing a strong baseline; however, future studies may consider older mice. Female mice may have differential cytoprotection upon TRAF2 activation and therefore may show different effects. Here, we were able to establish enhanced cardiac reserve yet reduced global cardiac function. However, future studies should also consider the mechanism by which this protection is conferred and if this protection is sustained across the life span in both male and female mice. Previous studies have found that TRAF2 restores mitophagy, which may be involved with the cytoprotection it provides. While these studies utilized terminal animals, they still provide strong evidence in live model methods. To further understand alterations in echocardiography in live animals, pressure-volume (PV) loops may be needed to study real-time cardiac systolic and diastolic and function.

## Conclusions

Previous studies suggest that TRAF2 overexpression may confer cytoprotection of the heart and perhaps afford a potentially important therapeutic target for CVD treatment. However, examination of the global cardiovascular function revealed a diminished cardiac function in the mice with TRAF2 overexpression with no differences in the aortic impedance implying no increased afterload. The diminished cardiac function in these mice and the apparent tolerance to ischemic insults may suggest that their cardiovascular reserve may be enhanced. Therefore, the roles of TRAF2, as well as potential downstream targets of NF-*κ*B, may be further elucidated to evaluate if they may be utilized as suitable therapeutic targets. The observations reported by us, and others were from findings in 2–3 month old mice and perhaps studies need to be conducted at older ages to determine if the cardioprotective effects of TRAF2 overexpression are maintained across life span without significant deterioration of cardiac function.

## Data Availability

The raw data supporting the conclusions of this article will be made available by the authors, without undue reservation.
