# Supplementary material for: Cardiovascular hemodynamics in mice with tumor necrosis factor receptor—associated factor 2 mediated cytoprotection in the heart
Source: Front Cardiovasc Med. 2023 May 9;10:1064640. doi: 10.3389/fcvm.2023.1064640 (PMC10203617; doi:10.3389/fcvm.2023.1064640)
Supplement: Supplementary file 1 [file Image1.pdf]

S. Figure 1

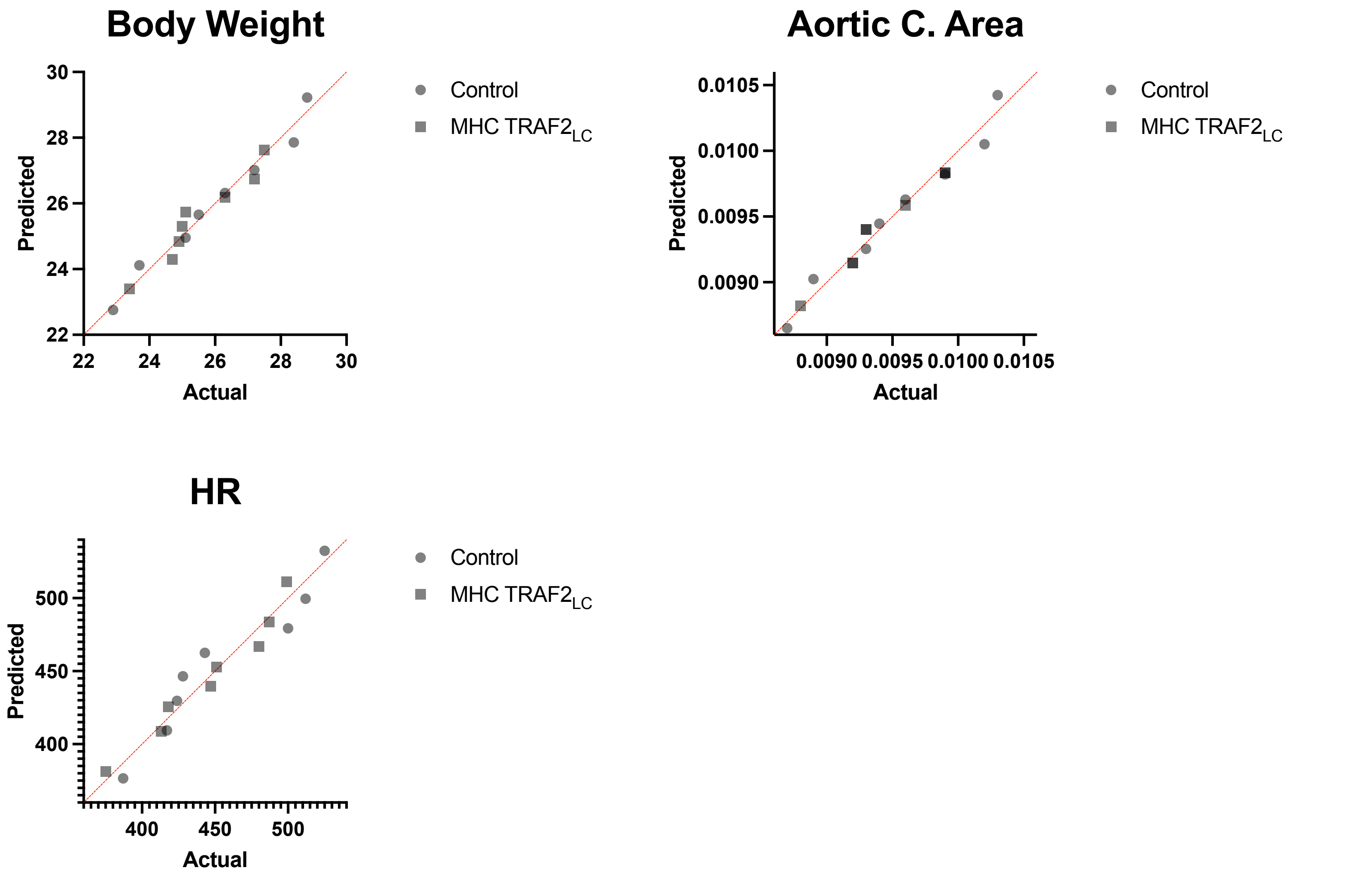
Body Weight
Predicted
Actual
22
24
26
28
30
Control
MHC TRAF2LC
Aortic C. Area
0.0090
0.0095
0.0100
0.0105
HR
400
450
500

S. Figure 2

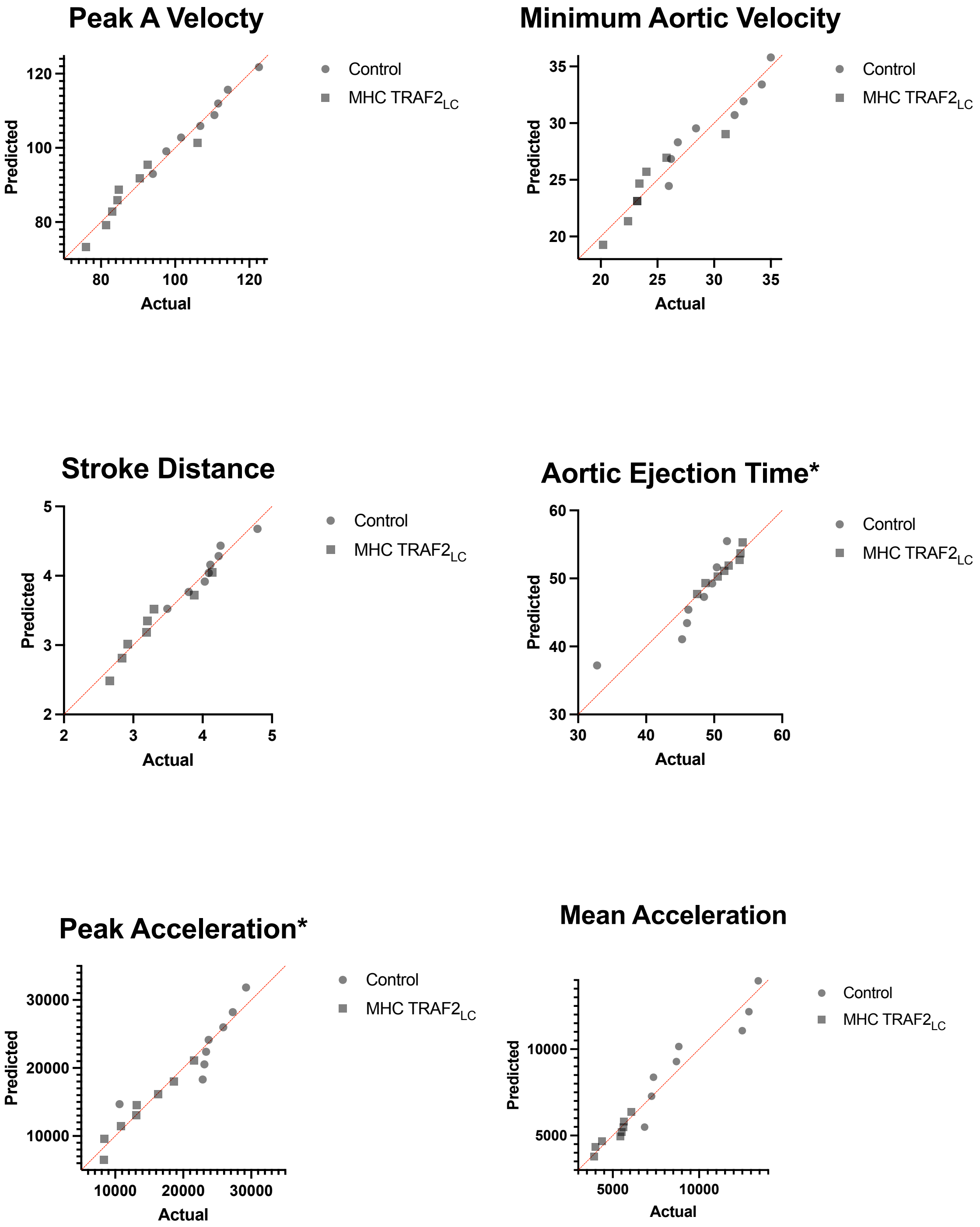

Peak A Velocty
Minimum Aortic Velocity
Stroke Distance
Aortic Ejection Time*
Peak Acceleration*
Mean Acceleration
Predicted
Actual
Control
MHC TRAF2$_{LC}$

S. Figure 3

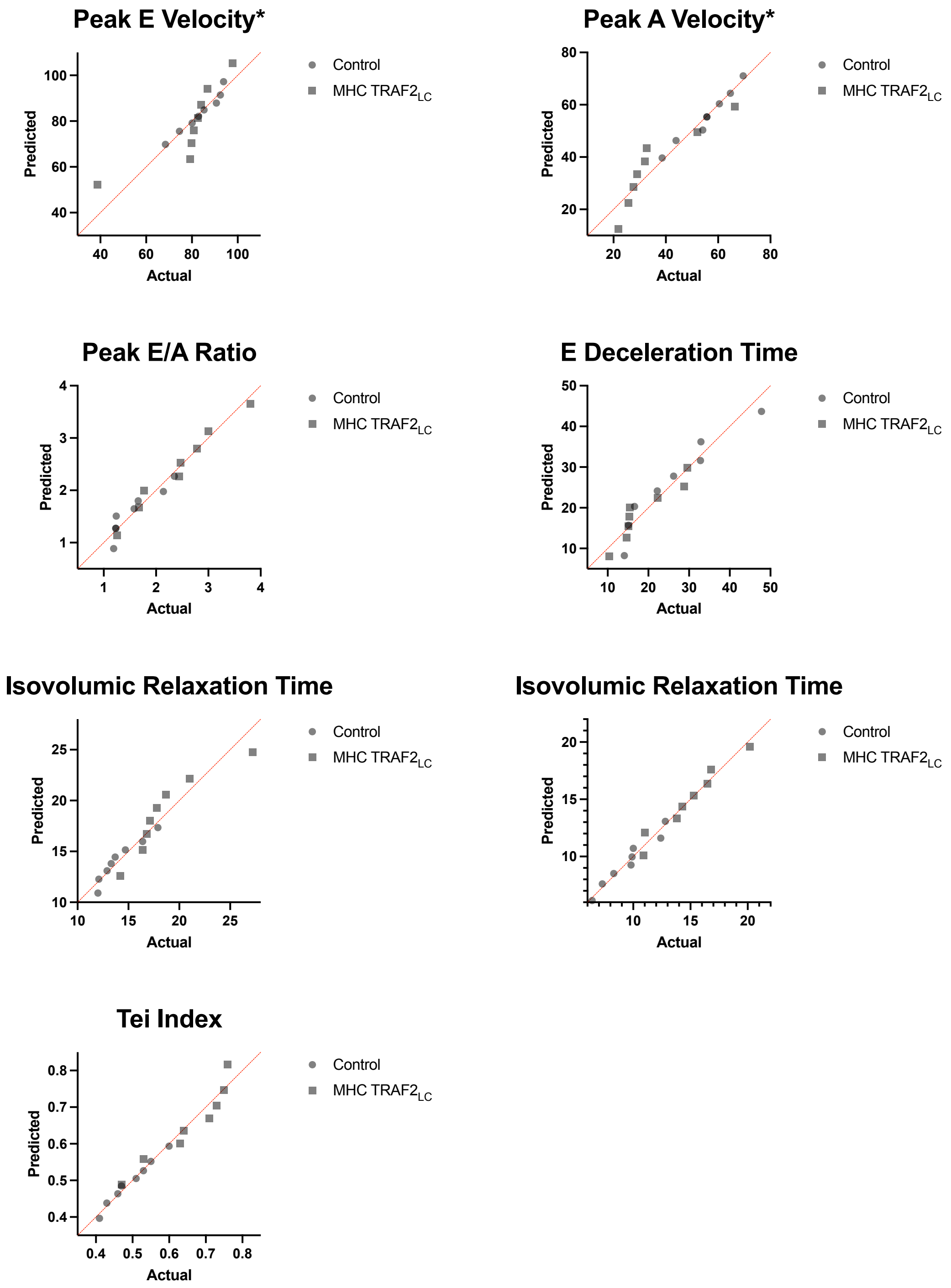

Peak E Velocity*
Predicted
Actual
Control
MHC TRAF2LC
Peak A Velocity*
Predicted
Actual
Control
MHC TRAF2LC
Peak E/A Ratio
Predicted
Actual
Control
MHC TRAF2LC
E Deceleration Time
Predicted
Actual
Control
MHC TRAF2LC
Isovolumic Relaxation Time
Predicted
Actual
Control
MHC TRAF2LC
Isovolumic Relaxation Time
Predicted
Actual
Control
MHC TRAF2LC
Tei Index
Predicted
Actual
Control
MHC TRAF2LC

S. Figure 4

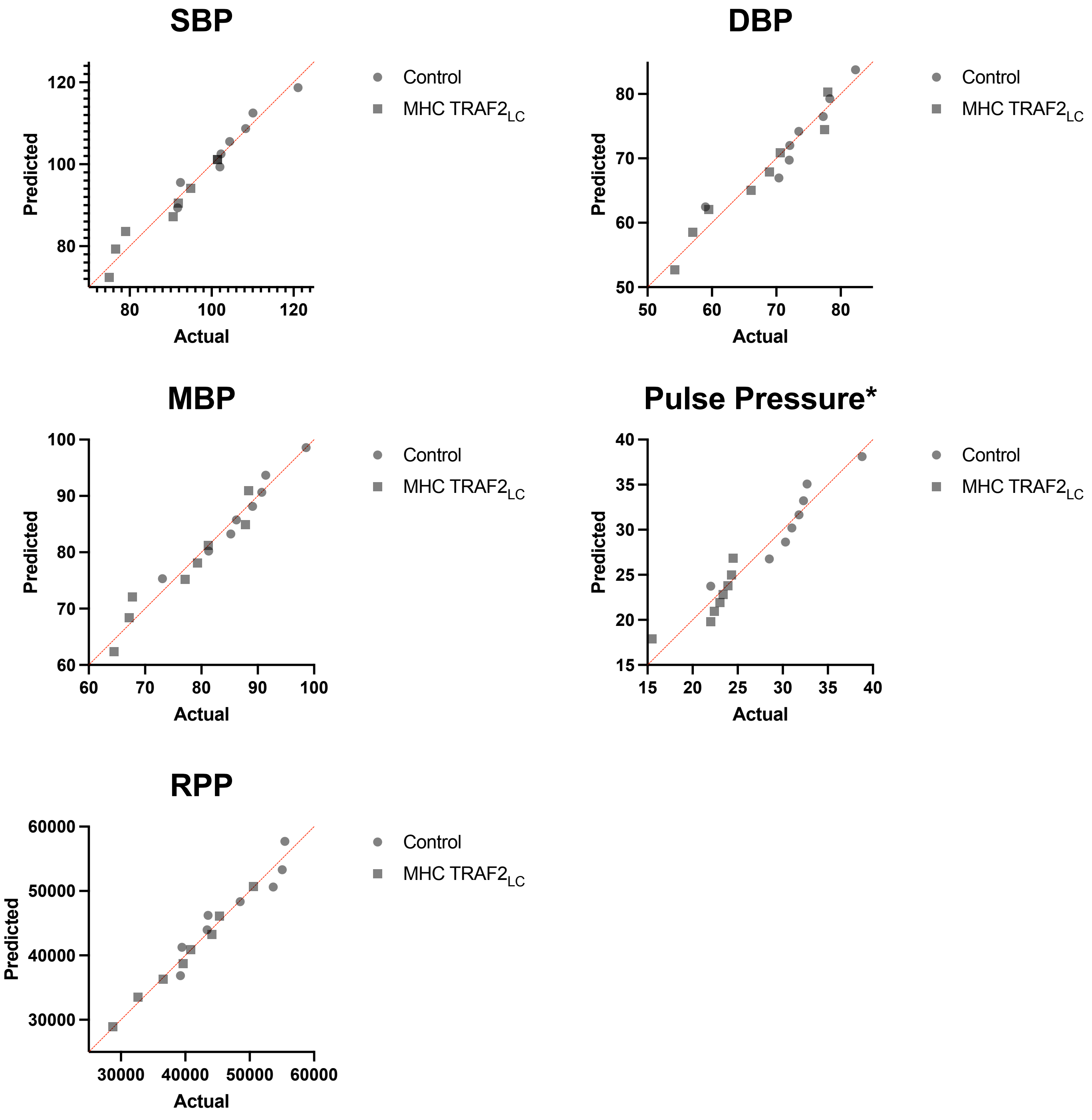
SBP
DBP
MBP
Pulse Pressure*
RPP
Predicted
Actual
Control
MHC TRAF2LC
120
100
80
50
60
70
90
15
20
25
30
35
40
30000
40000
50000
60000

S. Figure 5

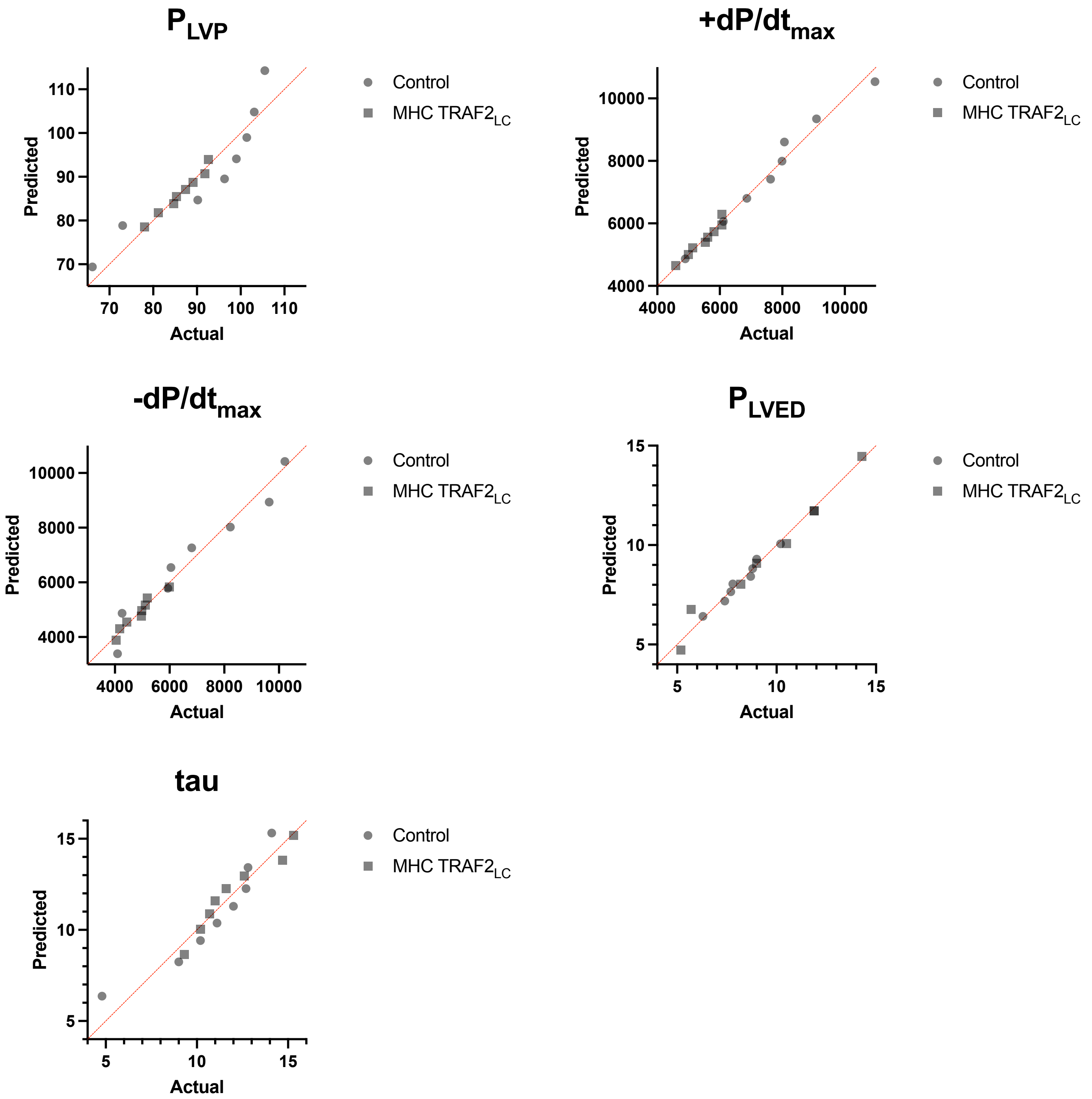
P_LVP
+dP/dt_max
-dP/dt_max
P_LVED
tau
Predicted
Actual
Control
MHC TRAF2_LC
70
80
90
100
110
4000
6000
8000
10000
5
10
15

S. Figure 6

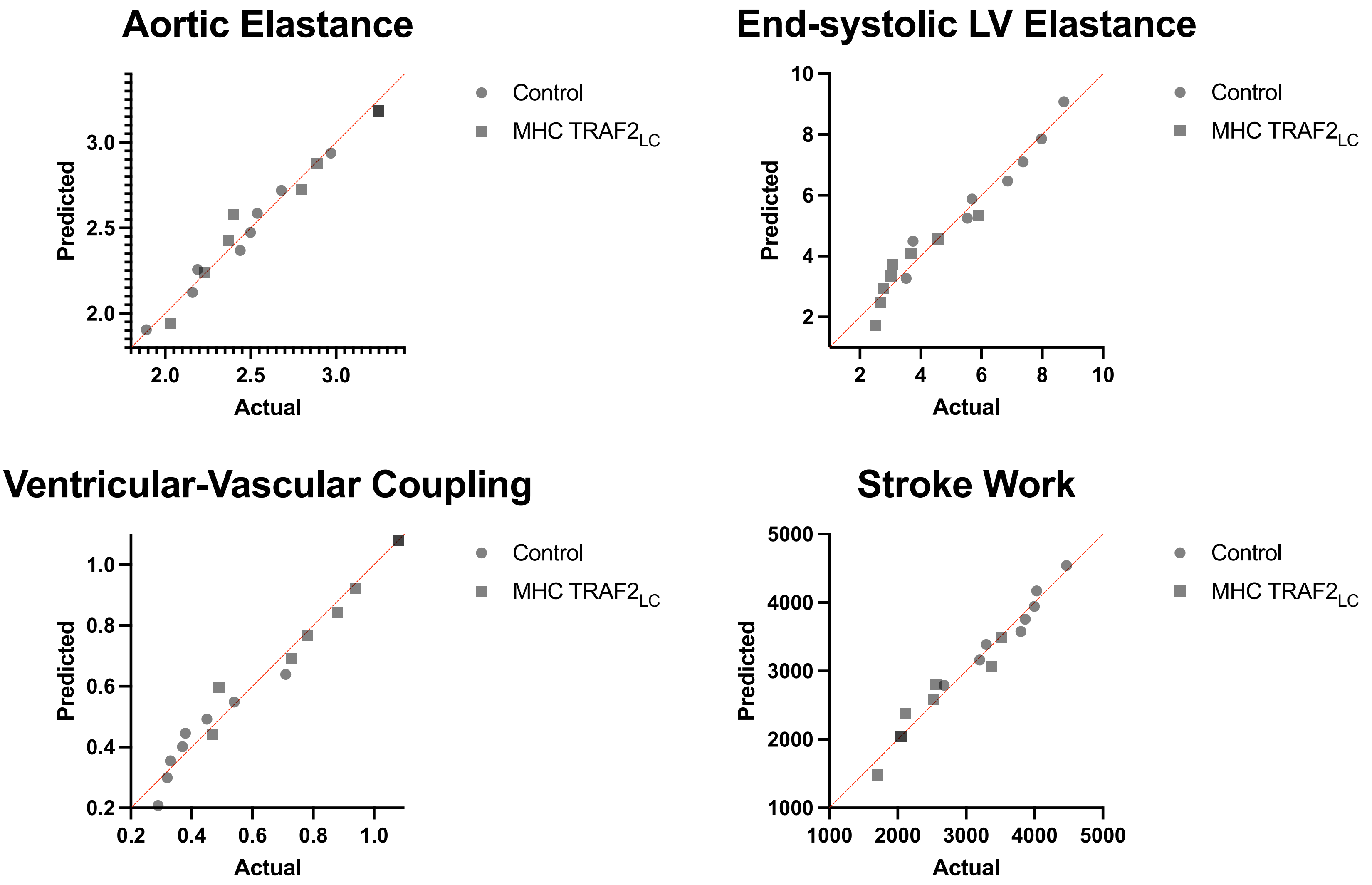

Aortic Elastance
Predicted
Actual
2.0
2.5
3.0
Control
MHC TRAF2$_{LC}$
End-systolic LV Elastance
Predicted
Actual
2
4
6
8
10
Control
MHC TRAF2$_{LC}$
Ventricular-Vascular Coupling
Predicted
Actual
0.2
0.4
0.6
0.8
1.0
Control
MHC TRAF2$_{LC}$
Stroke Work
Predicted
Actual
1000
2000
3000
4000
5000
Control
MHC TRAF2$_{LC}$

S. Figure 7

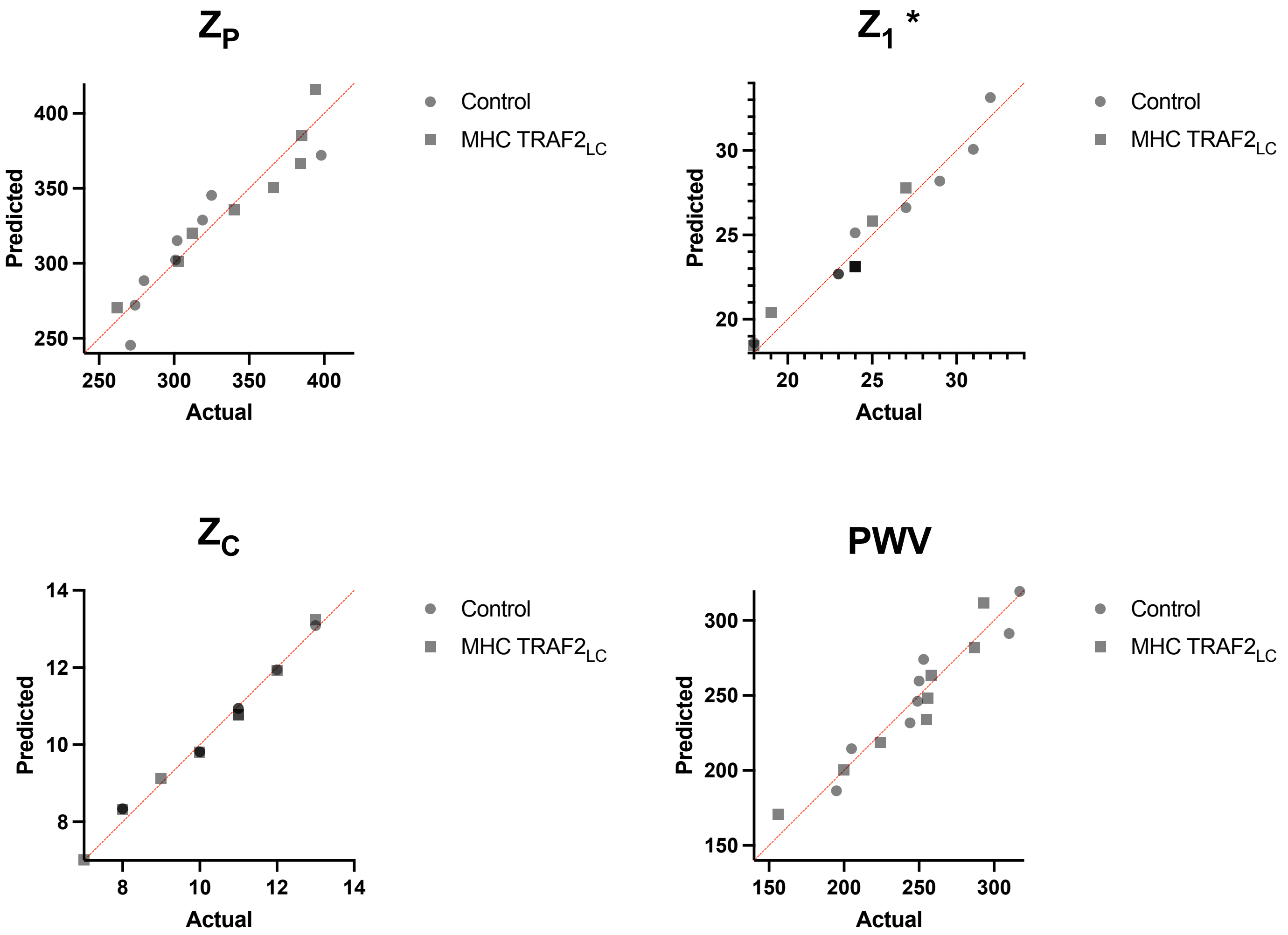

Z_P
Predicted
Actual
400
350
300
250
250
300
350
400
Control
MHC TRAF2_LC
Z_1 *
Predicted
Actual
30
25
20
20
25
30
Control
MHC TRAF2_LC
Z_C
Predicted
Actual
14
12
10
8
8
10
12
14
Control
MHC TRAF2_LC
PWV
Predicted
Actual
300
250
200
150
150
200
250
300
Control
MHC TRAF2_LC
